# The necessity and challenges of human papillomavirus testing for men

**DOI:** 10.3389/fcimb.2025.1563499

**Published:** 2025-06-30

**Authors:** Wenbo Ren, Yuting Jin, Lin Shi, Haizhen Chen, Zichen Zhang, Yifei Wang, Taiyu Zhai, Jing Huang

**Affiliations:** ^1^ Department of Clinical Laboratory, The First Hospital of Jilin University, Changchun, China; ^2^ College of Medical Technology, Beihua University, Jilin, China

**Keywords:** human papillomavirus, men, cross-infection, detection, challenges

## Abstract

Beyond its historical association with cervical cancer in women, human papillomavirus (HPV) infection poses a widespread health concern, particularly for men. Recent studies have highlighted the prevalence of HPV-related cancers, including penile and anal cancers, among the male population, making the case for male HPV testing all the more compelling. The potential for cross-infection between sexual partners underscores the need for comprehensive screening strategies. However, challenges still remain, such as the limited availability of testing methods and the absence of uniform medical guidelines, hampering effective HPV detection in men. Addressing these challenges through intensified research efforts and the revision of medical guidelines is crucial to enhancing prevention strategies and unlocking significant public health benefits.

## Graphical Abstract

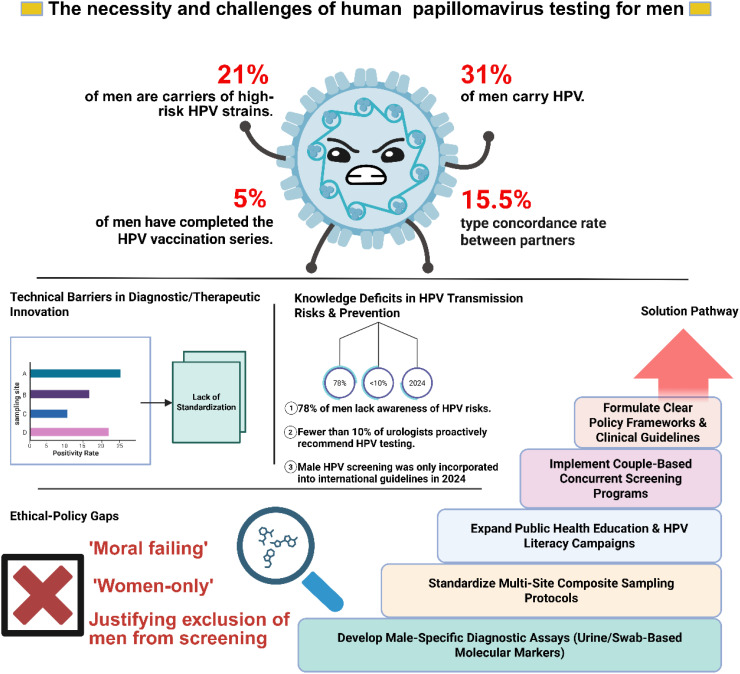

## 1 Introduction

Human Papillomavirus (HPV) is a globally prevalent virus primarily transmitted through sexual contact. HPV induces carcinogenesis through multiple molecular mechanisms, with the integration of viral DNA into the host genome being a pivotal event. This integration not only leads to the sustained overexpression of the viral oncoproteins E6 and E7 but also activates host oncogenes by generating HPV - human fusion transcripts. E6 and E7 disrupt cell cycle regulation and apoptosis by targeting and degrading the tumor suppressor proteins p53 and pRb, respectively (Harden and Munger, 2017; Mittal and Banks, 2017). Furthermore, E6 can independently affect the mitotic kinesin CENP-E, resulting in chromosome misalignment and genomic instability (Cosper et al., 2023). Additionally, HPV modulates the host immune response to establish an immunosuppressive microenvironment, and its persistent infection, coupled with immune evasion strategies, drives the gradual transformation of normal epithelium into invasive cancer (Liu et al., 2023).

Historically, research has predominantly focused on the association between HPV infection in women and cervical cancer, leaving a significant gap in understanding HPV’s impact on men. However, recent medical advancements have highlighted the severe health risks posed by HPV infection in men, including 50% of penile, 88% of anal, and 31% of HPV - associated oropharyngeal cancers, albeit with lower incidence rates (Saraiya et al., 2015; Malagon et al., 2024). The substantial public health burden associated with these cancers cannot be ignored. Globally, in 2018, it is estimated that HPV was responsible for approximately 620,000 new cases of cancer in women and 70,000 new cases in men (de Martel et al., 2020). The latest 2023 research report reveals that approximately one in three males aged 15 and above harbors at least one type of genital HPV, with a notable proportion (one in five) being infected with one or more high - risk oncogenic HPV types (Bruni et al., 2023). Emphasizing the significance of including males in strategies aimed at mitigating HPV infections and subsequently reducing the prevalence of HPV - associated illnesses is of utmost importance.

Notably, within marital or sexual partner relationships, the issue of HPV cross - infection becomes particularly pertinent, and such occurrences are frequent (Widdice et al., 2013). If one partner is infected with HPV, the other is highly susceptible to acquiring the virus through sexual contact without proper protective measures. Additionally, the sharing of intimate items such as towels and toilets can also facilitate HPV cross - infection through skin or mucosal injuries, further increasing the risk (Markowitz et al., 2014). This cross - infection poses a significant threat, as it can lead to the development of conditions like condyloma acuminata and cervical squamous intraepithelial lesions and even increase the likelihood of malignancies such as cervical cancer and oral cancer (Egawa N, 2023). Alarmingly, research indicates that nearly half of asymptomatic men harbor genital HPV infections (45.2%) (Lee et al., 2002). This significantly increases the risk of their spouses or sexual partners developing malignancies like cervical cancer. Therefore, the significance of male HPV testing extends beyond mere personal health management. It is crucial for the prevention and control of HPV cross - infection between spouses, as early detection and timely intervention can effectively mitigate the risk of HPV transmission, thereby safeguarding the overall well - being of both parties.

Despite the unequivocal importance of male HPV testing, numerous challenges persist in its practical implementation. Firstly, societal awareness surrounding male HPV infection remains limited. Many men mistakenly believe that HPV infection is exclusively a female concern, overlooking the potential risks they themselves may be exposed to. Secondly, the availability of testing methods for male HPV is relatively scarce, and their accuracy remains a concern. Notably, recent reports indicate that there are no clearly approved clinical testing methods specifically designed for male HPV infection, including in countries like China and the United States (Vives et al., 2020; Branch of Cancer P et al., 2023). However, an increasing number of researchers have begun to recognize this significant public health gap. In the 2024 guidelines on anal cancer issued by the United States, the role of males in the incidence of anal cancer was specifically highlighted, and early detection through HPV (human papillomavirus) testing was recommended. The ongoing reinforcement of this message is crucial to efforts to include males in HPV screening and cancer detection guidelines (Stier et al., 2024). Given these challenges, the aim of this review is to underscore the significance of male HPV testing and to urge for increased research efforts towards the development and enhancement of male-specific HPV testing methods. The progress in male HPV screening holds paramount significance in the prevention of cervical cancer among women.

## 2 It is necessary for men to undergo routine HPV screening

**Figure 1:**
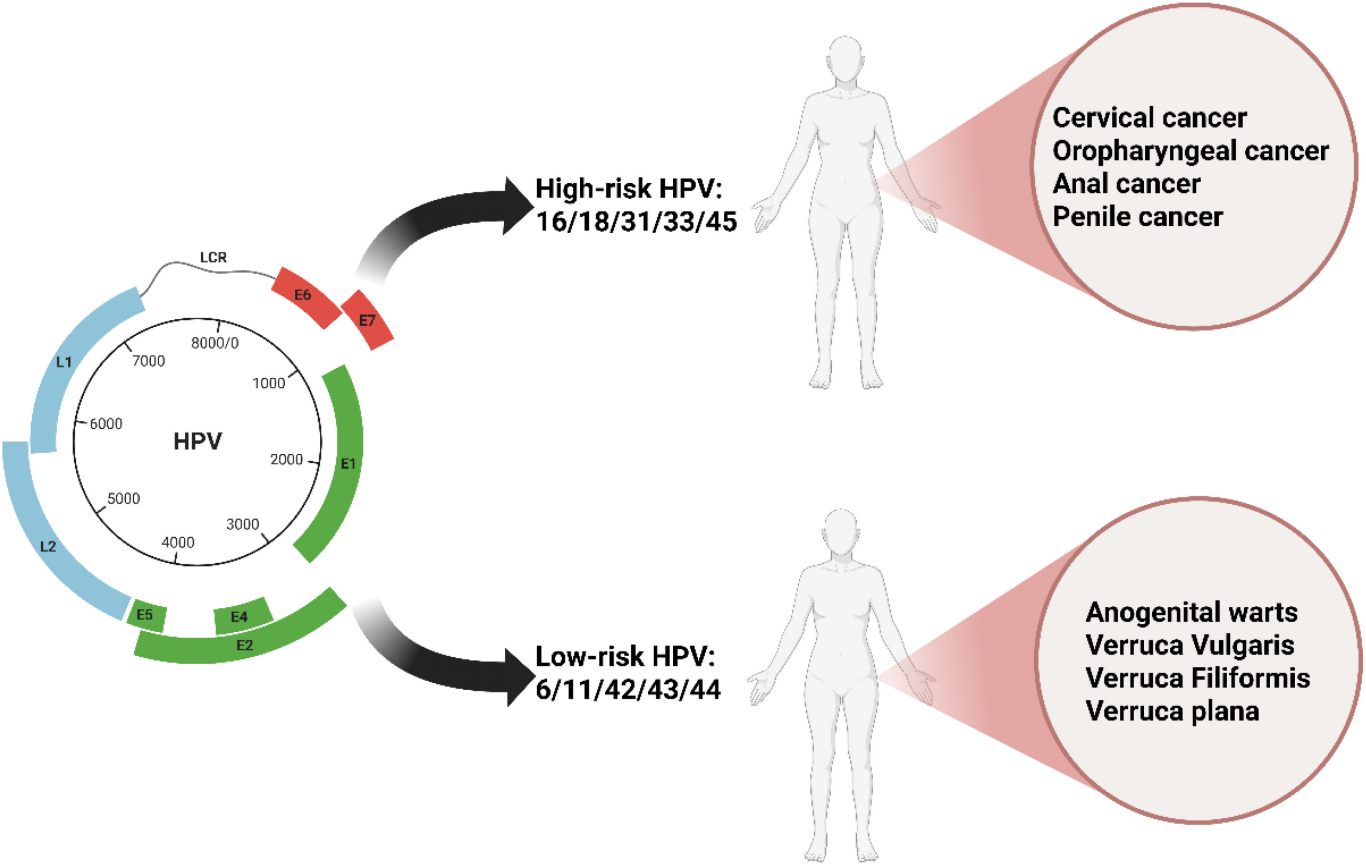
The fundamental characteristics, major clinical classifications, and associated diseases of HPV.

HPV is a primary cause of sexually transmitted infections. HPV belongs to the Papillomaviridae family and exists as a non-enveloped icosahedral virion. Its capsid is composed of structural units formed by 72 L1 protein pentamers, along with a small amount of the minor capsid protein L2 (Chen et al., 2023)。The viral genome is a circular double-stranded DNA molecule approximately 8 kb in length. It persists as an episome within the host cell, binding to host histones to form a chromatin-like structure. HPV is classified into different genera, including α, β, γ, δ, and μ, based on genomic variations. The α genus encompasses high-risk types such as HPV16 and HPV18, which are strongly associated with the development of malignant tumors like cervical cancer and oropharyngeal cancer (**Figure 1**). HPV demonstrates strict host and tissue specificity, infecting only undifferentiated epithelial cells in the basal layer of human skin or mucosa. The HPV genome can be divided into three functional regions. The early region (E region) encodes the E1 - E7 proteins, which play a crucial role in regulating viral replication. For example, the E6 and E7 proteins promote cellular malignant transformation by degrading the tumor suppressor proteins p53 and Rb, respectively. The late region is responsible for expressing the capsid proteins L1 and L2. The long control region contains the origin of replication and transcriptional regulatory elements. The replication cycle of HPV is closely linked to the differentiation process of host epithelial cells. After the virus enters basal cells through micro-abrasions, the early genes are activated, initiating low-copy replication. As the cells differentiate towards the surface layer, the E6 and E7 proteins override cell cycle checkpoints, driving the extensive amplification of the viral genome. Eventually, in terminally differentiated granular layer cells, the late genes are expressed, and progeny virions are assembled. These virions are then released through epithelial desquamation, completing the transmission cycle (Malik et al., 2023). This dependence on cellular differentiation often results in a latent state of HPV infection, presenting challenges for clinical detection and intervention.

**Figure 2:**
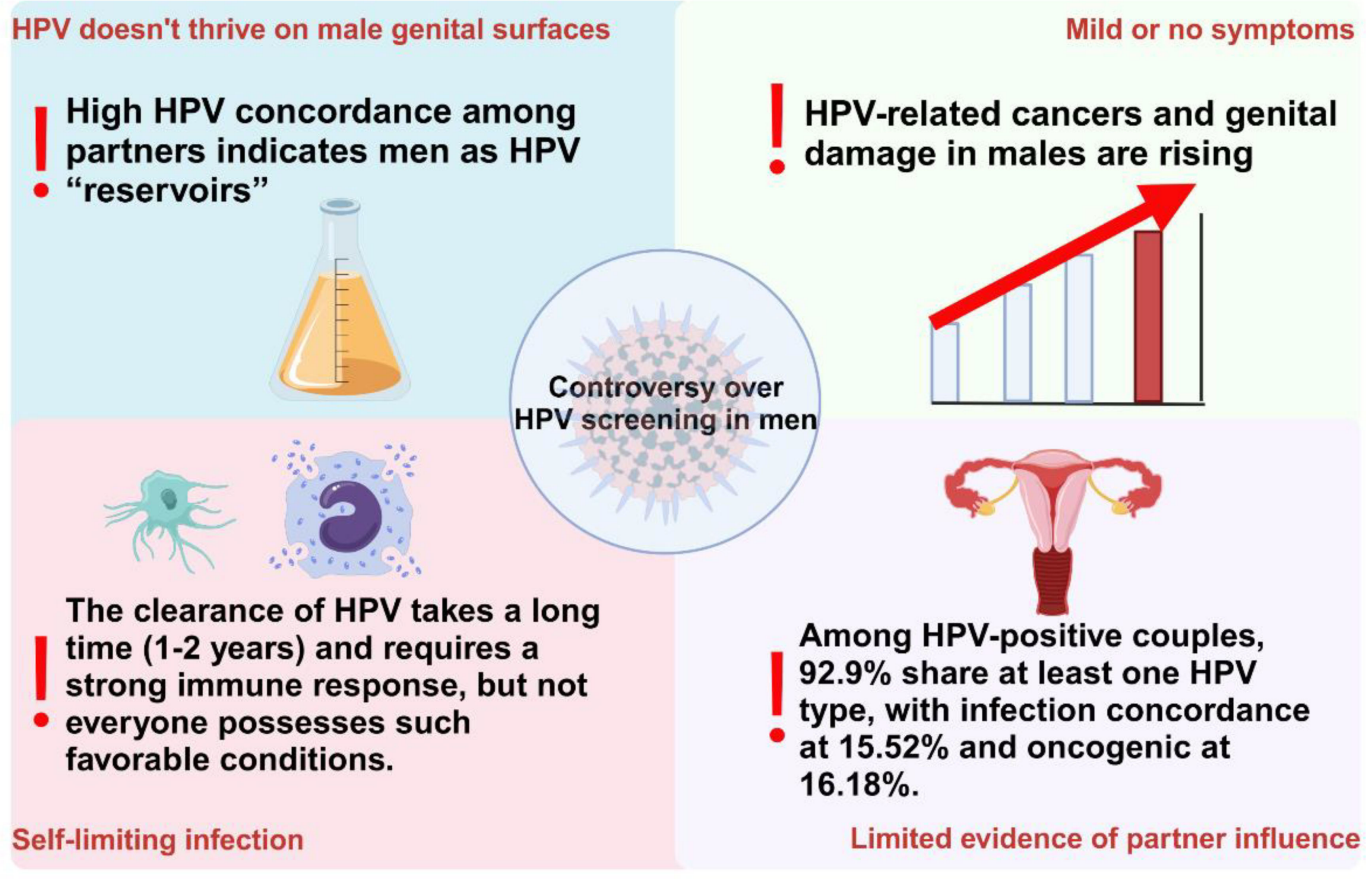
The four main points of controversy regarding the implementation of HPV screening for males currently.

Recent studies have shown that, depending on the population sampled, geographic region, detection method, and specific anatomical site or sample type, the prevalence of HPV among men ranges from 3.5% to 45%, which is slightly higher than the 2% to 44% prevalence among women (Kombe Kombe et al., 2020). Notably, approximately one - quarter of men carry high - risk HPV types (Lee et al., 2002; Das et al., 2023). These data highlight the significance of HPV screening for men. However, the question of whether routine HPV screening should be implemented in men remains contentious. HPV screening for men is not currently recommended for four main reasons: the absence of approved testing methods (Workowski et al., 2021; Branch of Cancer P et al., 2023); the limited survival of the virus on male genitalia; the self - limiting nature of most infections (Kreisel et al., 2021); and insufficient evidence regarding the role of sexual partners in virus persistence or transmission (Vives et al., 2020). These reasons collectively illustrate the complexities and uncertainties associated with implementing HPV screening in men (**Figure 2**). Consequently, it is imperative that we continually reassess and update our perspectives on this issue, based on the latest research findings, to ensure the scientific rigor and effectiveness of public health policies.

### 2.1 The incidence of HPV-related cancers in men warrants significant attention

Previously, it was widely believed that HPV is naturally prevalent and thrives in damp environments. However, this characteristic somehow renders it less capable of surviving and proliferating effectively on the surfaces of male genitalia. Furthermore, HPV infections are often characterized by their self-limiting nature, where a robust immune system can promptly eliminate the virus, thereby reducing the necessity for routine screening (Dunne et al., 2006). Nevertheless, it appears that the harm imposed on males during the persistence of HPV has been underestimated.

For instance, HPV positivity in semen can adversely affect sperm quality, potentially compromising reproductive capabilities (Garolla et al., 2013). Adding to this concern, the clearance of HPV frequently requires extended periods (1–2 years) and robust immunological responses. Unfortunately, not all individuals possess such favorable conditions. Persistent infections with high-risk HPV strains, especially those lasting over 2 years, significantly increase the risk of carcinogenesis (Barzon et al., 2010). This includes not only cervical cancer in females but also anal cancer, penile cancer, and HPV-associated oropharyngeal cancers in males (Suk et al., 2018; Cuschieri et al., 2021; Bruni et al., 2023).

Oropharyngeal cancer has the highest HPV attribution ratio (31%) among global head and neck cancers, and this malignancy is closely associated with HPV-16 infection (Malagon et al., 2024). In the demographic breakdown of HPV-associated oropharyngeal cancer cases, males significantly outnumber females (Marur et al., 2010). Penile cancer is a rare type of malignant tumor with an incidence rate of approximately one in 100,000 (Sachdeva et al., 2024). The incidence rate in South America and parts of Southeast Asia is significantly higher, accounting for 1 - 2% of male malignant tumor cases, and it is even higher in some parts of Africa, such as Uganda (Brouwer et al., 2023). A recent study showed that there were approximately 36,068 new cases of penile cancer and 13,211 deaths in 2020 (Fu et al., 2022). Moreover, the incidence of penile cancer is still slowly rising, which may be attributed to the increasing HPV infection rate (Qi et al., 2020). Similarly, anal cancer is another HPV-driven (88%) malignancy. In the past few decades, the overall incidence of anal cancer has been rising, particularly among male individuals engaging in high-risk sexual activities within the homosexual community (Malagon et al., 2024). Although anal cancer is relatively rare, studies have shown that not only the incidence but also the mortality rate of anal cancer is increasing in the United States (Holliday et al., 2023). It is noteworthy that when discussing this finding, the author mentioned that these data may reflect the growing prevalence of immunosuppressed adults in the United States. One hypothesis is that “comorbidities and iatrogenic immunosuppression may have the greatest impact on adults or the elderly, impairing HPV clearance and immune cancer surveillance.” This challenges the previously held belief that HPV can be naturally cleared by one’s own immune system. Although the incidence rates of penile cancer and anal cancer are relatively low, a low incidence rate does not seem to be a valid reason to overlook disease prevention.

These data incontrovertibly underscore a dire and pressing reality: the risk posed by HPV to men’s health is escalating, particularly among immunocompromised individuals, where the vulnerability is heightened. This revelation underscores the urgent need to address a long-standing oversight—the paramount significance of HPV screening in men. Historically, the discourse surrounding HPV and its associated diseases has been predominantly centered on women, particularly in the context of cervical cancer prevention and screening. However, as our understanding evolves and data accumulates, it becomes evident that HPV poses a formidable threat to men’s health as well, with penile and anal cancers serving as prominent examples. Consequently, this paradigm shift necessitates a critical reassessment and refinement of our strategies for implementing HPV screening in men, ensuring that they are commensurate with the evolving scientific understanding and the pressing health needs of this population.

### 2.2 Men occupy a pivotal position in the transmission of HPV

In the past, there was a significant limitation in HPV screening for men, primarily due to the lack of definitive evidence supporting their specific role in the persistence of the virus or its transmission between partners (Vives et al., 2020). However, recent research has highlighted a crucial fact: men play a pivotal role in protecting their partners from HPV-related cancers, particularly cervical cancer (Lin et al., 2022; Zou et al., 2022). This revelation not only broadens our understanding of HPV infection and its consequences but also underscores the importance of gender-inclusive health management, emphasizing the need for increased attention to HPV-related health issues in men.

Large-scale studies have emphasized the critical role of men in protecting their partners from HPV-associated malignancies. A notable 2015 study involving 900 heterosexual couples found a 15.52% concordance rate for shared HPV infections, with oncogenic types showing an even higher rate of 16.18%. This suggests that HPV transmission between partners is common, though not inevitable. The study introduced the concept of an “HPV transmission window” to define and quantify the risk period for transmission, revealing a higher risk for male-to-female transmission, especially of oncogenic HPV types (Liu et al., 2015).

Given physiological differences, women are more susceptible to oncogenic HPV infections, which, if undetected and untreated, can progress to precancerous lesions and ultimately lead to cancers of the cervix, vagina, vulva, anus, and oropharynx (de Martel et al., 2020). A recent study in a Chinese city examined HPV infection rates among heterosexual couples, revealing significantly elevated infection rates in women across all HPV types compared to men. The HPV concordance rate among these couples was 15.5%, consistent with prior research. Notably, among HPV-positive couples, 92.9% shared at least one common HPV type. Male genital areas, including the penis, glans, and coronal sulcus, exhibited concordance rates of 20.0%, 21.8%, and 14.9% with female vaginal, vulvar, and perianal/anal areas, respectively, which were higher than those in control groups. These findings suggest that the male genitalia serve as a primary source of HPV transmission to women, while women can also transmit HPV to men through vaginal, vulvar, and perianal/anal regions. Additionally, factors such as younger age, frequent sexual activity, multiple sexual partners, or persistent HPV infection in one partner significantly increase the risk of HPV transmission between spouses (Su et al., 2019). Furthermore, numerous previous studies have provided evidence for the mutual transmission of HPV between partners (Burchell et al., 2010; de Lima Rocha et al., 2012).

Current guidelines clearly state that specialized HPV screening for men is not particularly emphasized, given that most male HPV infections, including high-risk types, lack significant clinical symptoms (Gamboa-Hoil, 2023). However, this position seems to overlook the crucial role men play in the HPV transmission chain and the potential data gaps resulting from current screening limitations.

It is important to recognize that even though male HPV infections often do not present symptoms, they can still pose long-term health risks, including serious complications and fatal diseases (Sung et al., 2021). Recent scientific advancements have highlighted the significant role male genitalia play in transmitting HPV to women. Combined with the high infection rates and various high-risk factors within the male population, this underscores the urgent need for HPV screening in men. Current observations are based on limited detection capabilities, suggesting that the actual prevalence of HPV in men might be more severe than existing data indicates.

Given this, it is necessary to reassess the need for HPV screening in men. Routine HPV screening for men would not only facilitate the early identification and effective management of infections but also reduce the incidence of HPV-related malignancies in women, thereby significantly enhancing public health. Particularly with high-risk HPV infections, such a screening strategy is crucial. It protects individual health and contributes positively to the overall health and well-being of society. Therefore, incorporating routine HPV screening for men into public health planning is a scientific, rational, and urgent public health measure.

## 3 The challenges facing male HPV testing

**Figure 3:**
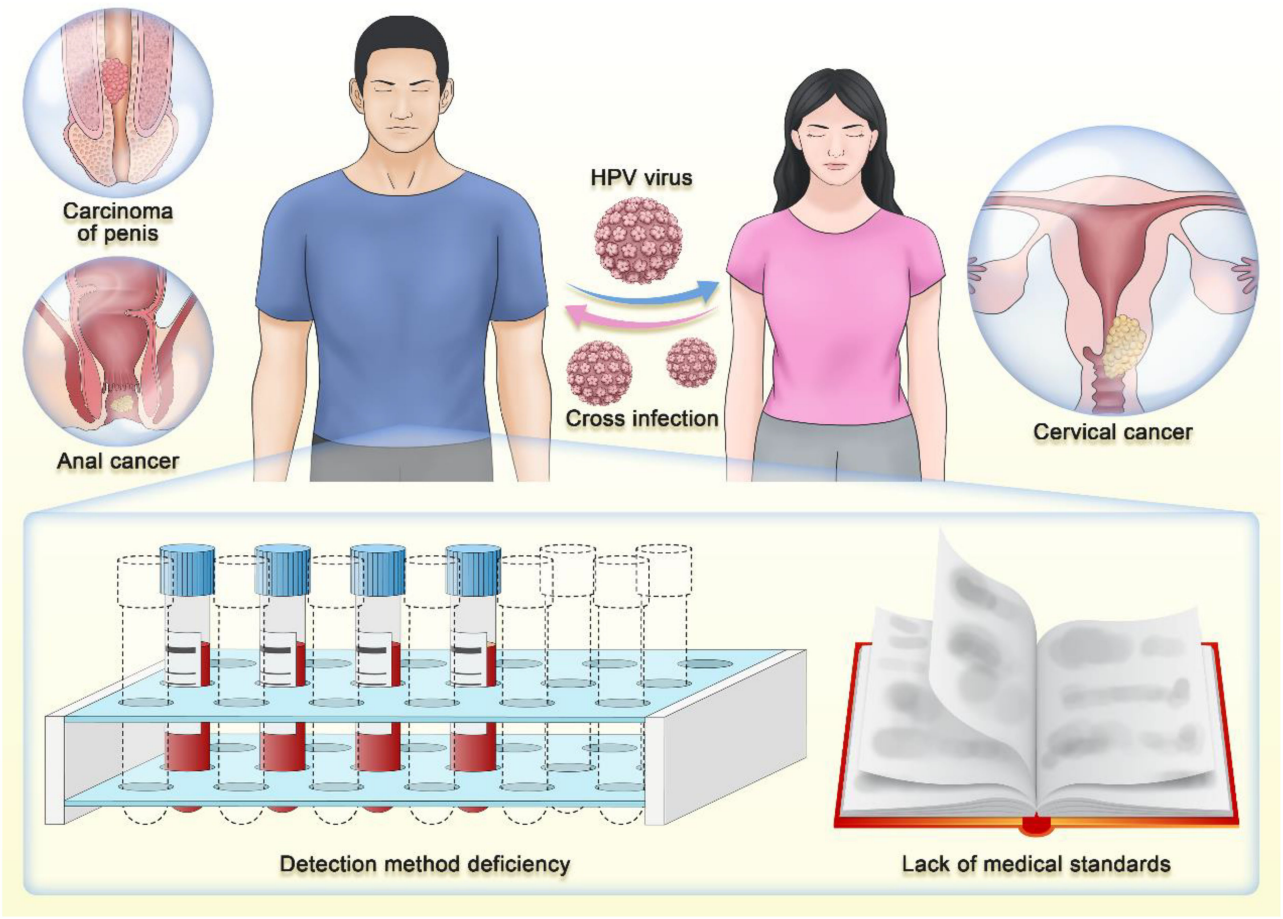
The current challenges encountered in conducting HPV testing for men.

A recently published meta-analysis has underscored the high prevalence of high-risk HPV among men in most regions, with rates ranging from 20% to 30%. It also highlighted the need to strengthen HPV prevention as part of comprehensive sexually transmitted infection control measures. Furthermore, the study emphasized the scarcity of HPV data among men in some regions of the world and the importance of expanding HPV prevalence surveys in these areas to evaluate the effectiveness of prevention strategies (Bruni et al., 2023). This further supports our argument that there is a need to enhance HPV screening among men, even if it is limited to high-risk HPV types. However, the challenges associated with male HPV screening are significant. The primary challenges include the lack of specialized HPV testing methods for men, the absence of clear medical guidelines to support this practice, and social barriers to including men in HPV screening programs (**Figure 3**).

### 3.1 Approved specialized HPV detection methods for males are currently unavailable

Our foremost challenge lies in the absence of dedicated screening methods for male HPV detection. This gap encompasses a comprehensive lack of standardized detection processes, inefficient sampling strategies, and limited awareness among healthcare professionals regarding male HPV screening. The typically asymptomatic nature of HPV in males has significantly hindered the development of standardized detection parameters (Chaturvedi et al., 2023; Deshmukh et al., 2023). The conventional strategy, which focuses on identifying clinical manifestations, often fails to detect HPV in men until distinct indicators such as genital warts or associated cancers are evident (Workowski et al., 2021). Consequently, HPV remains undiagnosed and continues to spread.

For women, direct detection methods primarily focus on identifying the presence of HPV DNA or RNA (Workowski et al., 2021). Currently, there are multiple clinically applicable methods for HPV screening and infection diagnosis in women. The HC2 method is sensitive and reliable, making it suitable for large-scale screening; however, it cannot differentiate among HPV subtypes and exhibits cross-reactivity issues (Xu et al., 2018). The Cervista method requires a small sample size, incorporates internal controls, but similarly cannot distinguish between subtypes and demonstrates cross-reactivity (Kurian et al., 2011; Boers et al., 2014). The AHPV method boasts high specificity, can reflect the transcriptional activity of the virus, and assess cancer risk; nevertheless, it is costly, unable to differentiate among HPV subtypes, and may produce false-negative results (Giorgi Rossi et al., 2022; White et al., 2024). The NGS method offers high throughput, can identify multiple HPV subtypes and mutations, and provides comprehensive genomic information; however, it is expensive and necessitates specialized equipment (Han et al., 2024; Pasquier et al., 2024). The ddPCR method is sensitive to low viral loads, allows for absolute quantification without reliance on a standard curve, and exhibits high specificity; however, it entails higher costs and requires specific equipment (**Table 1**) (Malin et al., 2021; Siravegna et al., 2022).

**Table 1:** The advantages and challenges of mainstream HPV testing methods for women.

Methods	Advantages	Challenges
HC2	Highly sensitive and reliable Suitable for mass screening	Difficulty in distinguishing between HPV subtypes Cross-reactivity issue
Cervista	Small sample size needed Internal controls included	Inability to specifically identify subtypes Potential for cross-reactivity
AHPV	Exhibits high specificity Reflects viral transcriptional activity Assesses cancer risk	Expensive cost Limitation in differentiating HPV subtypes Risk of false-negative outcomes
NGS	High throughput Identifies HPV subtypes/mutations Provides genomic information	High expense Need for specialized equipment
ddPCR	Detects low viral loads Absolute quantification, no standard curve	Higher costs Requires dedicated equipment

HC2, Hybrid Capture 2; AHPV, Aptima HPV assay; NGS, Next Generation Sequencing; ddPCR, Digital PCR.

The challenge in applying these HPV DNA tests in men stems from their sampling methods and efficacy. These tests, which mostly rely on cell samples from the cervix or vagina, are not feasible in males due to anatomical differences (Zou et al., 2022). Researchers have therefore explored alternative samples such as urine, oral fluid, or anal swabs for male HPV detection. However, the sensitivity and specificity of these alternatives may not match those of samples obtained from the cervix or vagina (D’Hauwers and Tjalma, 2009; Vorsters et al., 2012; Poljak et al., 2023).

The collection of these alternative samples also presents certain challenges. For instance, although urine samples are easier to collect, they may contain lower concentrations of HPV DNA, which diminishes detection sensitivity. Oral fluid samples offer the convenience of collection; however, ambiguous correlations with genital HPV infection affect their specificity (Poljak et al., 2023). It is noteworthy that this report does not specify the type of urine sample mentioned, whereas research utilizing first - void urine for HPV virus detection in men has demonstrated greater potential, thereby opening up new avenues for HPV testing and monitoring (Koene et al., 2016).

While anal swab samples, being in close proximity to male genital regions, may pose challenges in terms of comfort during collection, their value as a diagnostic tool necessitates further investigation into their stability and reliability (Nemcova et al., 2022). Appropriate sample handling and storage procedures are also critical, as improper practices may lead to the degradation of HPV DNA, reducing test accuracy (Vorsters et al., 2014). Optimizing sampling methods, enhancing sample sensitivity and specificity, and ensuring proper handling and storage practices are imperative for accurate male HPV DNA testing.

In **Table 2**, we provide an overview of the detection methodologies and corresponding positive rates employed in recent studies examining the prevalence of HPV infection among males. The existing data demonstrate that PCR has consistently been the predominant technology for HPV detection in both males and females over the past decade (Hebnes et al., 2015). However, the significant impact of anatomical sampling sites on detection outcomes in males warrants further investigation. While current studies predominantly employ multi - site swab sampling (e.g., coronal sulcus, urethral meatus), variations in viral load across anatomical regions may result in fluctuations in detection sensitivity, thereby complicating the interpretation of disparities in positivity rates. Although methodological comparisons between detection assays (e.g., PCR vs. HC2) have been conducted, the lack of standardized sampling protocols remains a critical issue. Future research should systematically compare detection efficiency across anatomical sites (e.g., coronal sulcus, penile shaft, urethral meatus) and prioritize the evaluation of the feasibility of non - invasive specimen types, such as first - void urine, to elucidate the mechanistic influence of sampling strategies on test results.

**Table 2:** Recent studies on HPV detection methodologies and positive rates in men.

Research Subject	Sample Type	Method	Positivity Rate	Reference
Non-sexually Transmitted Diseases males	30 specimens of circumcision, 110 swabs from the penis.	PCR	Patients with phimosis: 46.6%, Asymptomatic male penile swabs: 16.3%	(Afonso et al., 2016)
598 men and women in Alto Rio Solimo˜es, Amazonas state, Brazil	41 Men, 75 Women	The HC2 High-Risk HPV DNA Test	Men: 14.4%, Women: 24%	(Benzaken et al., 2012)
1551 men with a mean age of 35.86 ± 11.3 years	1392 balanopreputial, 435 urethral, 123 anal, and 67 condyloma lesions	PCR	Balanopreputial: 36.9%. Urethral: 24.9%. Mixed infection: 5.4%. Lesion swabs: 50.9%, Anal Swabs:49.6%, Condylomata: 71.4%	(Alvarez-Arguelles et al., 2013)
90 females and 117 males from a UK drop-in clinic offering integrated sexual health services	urine sample and comparator gold-standard sample (cervical liquid-based cytology sample or penile swab	PCR	Females: 59–67%, Males: 29–37%	(Cuschieri et al., 2011)
Males aged 14–59 years	penile swabs	PCR	42.2%	(Gargano et al., 2017)
340 Slovenian men from infertile couples	the external genitalia and semen	PCR	External genitalia: 37.12%, Semen samples: 13.61%	(Golob et al., 2014)
2460 Men in Denmark	Penile swab	HC2 test, and PCR	HC2 test: 22.2% PCR: 41.8%	(Hebnes et al., 2015)
213 Healthy male volunteers at VU University (Amsterdam, the Netherlands)	Penile scrape and semen samples	GP5+/6+ PCR and SPF10-PCR	Semen samples: 27%	(Luttmer et al., 2015)
262 Heterosexual men at the Provincial Sexually Transmitted Infection Clinic in Vancouver, Canada	Glans penis/foreskin, penile shaft (ventral and dorsal surfaces) and scrotum	Roche Amplicor HPV test, and Roche Linear Array HPV typing assay	Clinician-collected specimens: 69.8% Self-collected specimens: 55.3%	(Ogilvie et al., 2009)
1813 men in Tanzania	Penile samples	HC2 test and INNO-LiPA HPV Genotyping Extra test	20.5%	(Olesen et al., 2013)
504 clinically healthy heterosexual couples from four municipalities in the State of Mexico, Mexico	External male genitals, cervical samples	PCR	External male genitals: 20.5% Cervical samples: 13.7% Couples concurrently infected: 6.7%	(Parada et al., 2010)
HIV-uninfected 493 men and 500 women aged 18–22 years from the University of Botswana in Gaborone	Vaginal and penile swabs	PCR	Women: 63.0%, Man: 31.4%	(Ramogola-Masire et al., 2022)
752 men (mean age 32.4 years; median life-time sex partners 34) visiting urology outpatient clinics in St. Petersburg, Russia	Urethral swabs and Expressed Prostate Secretions samples	General primer PCR	47.9%	(Smelov et al., 2013)

PCR, Polymerase chain reaction; LiPA25system, Linear probe assay 25 system; HC2 test, Hybrid capture 2 HPV test; SPF10 DEIA, Serial plasma filter 10 dot enzyme immunoassay.

In conclusion, male HPV DNA testing presents multifaceted challenges concerning sampling methodologies and detection efficacy. Current research should not only focus on developing tailored screening technologies to optimize testing procedures but also urgently establish complementary clinical management protocols. Given the high prevalence of high - risk HPV infections in males, in contrast to the low progression to malignancy, differentiated follow - up strategies must be developed, including setting appropriate screening intervals for partner protection and determining viral load monitoring thresholds. Furthermore, interventions for HPV - positive individuals require transcending the limitations of current guidelines, such as by exploring the potential of topical immunomodulators for viral clearance in males. Future studies should synchronize technological development with the standardization of clinical pathways to establish a comprehensive prevention system that encompasses screening, intervention, and follow - up.

### 3.2 Lack of clear medical guidelines for support

One significant barrier to effective male HPV detection is the pronounced absence of established medical guidelines advocating for male HPV screening. This notable deficiency primarily stems from a historical bias in HPV research and policymaking, which has predominantly focused on women due to the direct association between HPV and cervical cancer (Ong et al., 2023). Notably, while most current HPV screening guidelines (e.g., the latest Chinese expert consensus and American Cancer Society recommendations) remain predominantly female-centric, the newly released International Guidelines for Anal Cancer Prevention have formally integrated male HPV testing into standardized screening protocols, representing a substantial advancement in gender-specific screening strategies (Fontham et al., 2020; Branch of Cancer P et al., 2023; Stier et al., 2024).

This gendered approach to HPV screening presents numerous challenges for successful HPV detection in men. Firstly, it overlooks the male contribution to overall HPV prevalence, thereby perpetuating the cycle of transmission. Globally, nearly one-third of men are infected with at least one type of genital HPV, and approximately one-fifth of men are infected with one or more high-risk/carcinogenic HPV types. In reality, HPV prevalence is high among men aged 15 and above (Bruni et al., 2023). Consequently, it is equally crucial to include the male population in comprehensive HPV prevention strategies.

Secondly, the lack of emphasis on male HPV screening minimizes the risk of male HPV-associated diseases, including oropharyngeal, anal, and penile cancer (Giona, 2022; Xu et al., 2024). Finally, in the absence of standard medical guidelines, healthcare professionals lack clear direction when communicating the importance of male HPV screening to their patients. This is evident from the vaccination rate of male HPV vaccines. Although HPV vaccines have been recommended for use in males since 2011, the vaccination rate among males has remained severely inadequate over the following 10 years. According to estimates, the global proportion of individuals who have completed the first dose and the full course of HPV vaccination in 2022 is as follows: among women within the target age range (generally 9–14 years old), 21% have completed the first dose and 15% have completed the full course of vaccination, while among men, 6% have completed the first dose and 5% have completed the full course. Among 15-year-old girls globally, the first-dose HPV vaccination rate is 21%, and the proportion of those who have completed the full two-dose course is 17%. For boys of the same age, the corresponding figures are 7% and 5% (Lei et al., 2020). The vaccination rate of male HPV vaccines is severely inadequate compared to women, which may be attributed to the significant lack of health knowledge dissemination. A survey conducted in 2019 among 4,000 female and 1,000 male individuals aged 18–45 in mainland China found that the awareness rate of HPV was 22% among men and 31% among women, while the awareness rate of HPV vaccines was 23% among men and 34% among women (Hu et al., 2021). The insufficient dissemination of health knowledge is a critical reason for this issue. Therefore, current health education requires dual advancements: not only must awareness of HPV screening and vaccination be enhanced among males, but risk education regarding HPV-associated cancers must also be systematically integrated. At the same time, governments, medical institutions, and various sectors of society should collaborate to develop and improve relevant policies and guidelines, providing clear direction and support for male HPV screening and vaccination. Due to the widespread implementation and popularization of HPV screening and vaccination programs targeted at women, the upward trend in cervical cancer incidence has been effectively curbed in numerous countries worldwide, marking a significant milestone in public health (Lin et al., 2021; Singh et al., 2023). However, the expanding understanding over the last decade of the role of HPV infection in other cancer types, such as oropharyngeal and anogenital cancers in both sexes, coupled with emerging data indicating an increasing incidence of these cancers, underscores the necessity for reevaluating the adequacy of current guidelines (Kombe Kombe et al., 2020; Scott-Wittenborn and Fakhry, 2021; Gormley et al., 2022). These concerning trends necessitate medical institutions to reassess and adjust their HPV screening guidelines to better include the male population. A recent meta-analysis has revealed that the overall prevalence rate of HPV among men is 31%, with a notable 21% prevalence rate for high-risk HPV types. The authors emphasize the importance of including men in comprehensive HPV prevention strategies to mitigate HPV-related morbidity and mortality among them, ultimately striving to eradicate cervical cancer and other HPV-related diseases (Bruni et al., 2023). We endorse this perspective and further suggest that the current epidemiological data on male HPV may be underestimated due to the limited availability of HPV detection methods tailored specifically for men, and many men remain unaware of the need for, and methods of, HPV testing.

Refining existing guidelines to include dedicated male HPV screening directives could aid in disrupting HPV transmission while highlighting the role of HPV in causing cancers within the male population. An inclusive HPV screening guideline could potentially reduce disease burden and have a broad health impact. To support these guideline modifications, it is essential to conduct more comprehensive research, including experimental studies and randomized controlled trials, to provide reliable evidence in favor of male HPV screening.

### 3.3 Men’s HPV screening faces multidimensional barriers

Incorporating men into HPV screening programs encounters multidimensional social barriers, which are deeply rooted in gendered disease perception frameworks and structural health inequalities. The public health sector has long portrayed HPV as a “women-only” health issue, and this gendered narrative creates a dual cognitive dilemma for male screening. On the one hand, there is a notable bias in the generation of medical knowledge, as HPV health education materials are typically designed exclusively for women, resulting in a widespread knowledge gap regarding the disease among men. On the other hand, disease stigmatization varies based on gender. In conservative cultural contexts, an HPV-positive status is considered a moral failing, and male patients often face stronger social condemnation. This stigmatization is further reinforced within the medical system, with clinical practice indicating that very few urologists proactively recommend HPV testing for male patients, revealing cognitive limitations within the professional community (Wendrich and Krabbenborg, 2021).

To overcome these social barriers, it is necessary to establish an interdisciplinary intervention framework. An information restructuring strategy can achieve destigmatization by emphasizing the skin-to-skin transmission attribute of HPV (which accounts for 40% of infections). Pilot projects have demonstrated that using “cancer prevention” messaging increased male participation rates by 57% (Piscatelli et al., 2024; Wolf et al., 2024). In terms of service system innovation, anonymous at-home self-testing kits can enhance screening rates. For example, the TakeMeHome project in the United States successfully reached previously untested populations by offering online ordering and self-collection for HIV and sexually transmitted infection tests, highlighting the crucial role of privacy protection mechanisms (Hecht et al., 2024). At the policy level, incentive-compatible mechanisms need to be implemented, such as linking corporate health management certification to male screening coverage, while revising cost-benefit assessment models to incorporate intergender externality benefits. The effectiveness of these intervention measures relies on a profound understanding of social structures, and only by dismantling the gendered nature of disease narratives can the transformation of HPV prevention and control from a biomedical model to a socio-technical paradigm be achieved.

## 4 Conclusion

It is reassuring to note that our concerns are not isolated. In recent years, several scholars have voiced their opinions in the academic arena. Specifically, two studies published in 2020 and 2022 highlighted the absence of male HPV detection methods and delved into the pivotal role of male HPV screening in preventing HPV infection and eradicating associated diseases (Vives et al., 2020; Zou et al., 2022). As our comprehension of HPV infection continues to evolve, there is an urgent imperative to fundamentally revamp screening strategies, particularly for male populations. In the past, owing to a predominant focus on female cervical cancer, the potential threat posed by HPV to male health has been overlooked for an extended period, underscoring the necessity of implementing comprehensive and systematic screening measures.

Given the incidence of HPV-related cancers in males and the risk of HPV transmission between sexual partners, it is imperative that we adopt more proactive and innovative measures in public health strategies. It is crucial to update the content of existing guidelines at appropriate junctures to underscore the significance of male HPV screening. Integrating male HPV testing into routine screening represents not only a vital step in safeguarding individual health but also a pivotal stride in fostering a shift towards a proactive prevention culture within society. This transformation will significantly expedite the development of male HPV screening methodologies, thereby augmenting the collective health resilience of our society and laying a robust foundation for the prevention and eradication of HPV-related diseases.
